# Calcaneal Spurs: A Potentially Debilitating Disorder

**DOI:** 10.7759/cureus.28497

**Published:** 2022-08-28

**Authors:** Vivek R Velagala, Namrata R Velagala, Tanishq Kumar, Arihant Singh, Ashok M Mehendale

**Affiliations:** 1 Medicine, Jawaharlal Nehru Medical College, Datta Meghe Institute of Medical Sciences, Wardha, IND; 2 Preventive Medicine, Jawaharlal Nehru Medical College, Datta Meghe Institute of Medical Sciences, Wardha, IND

**Keywords:** bony outgrowth, calcaneal tuberosity, gout, heel pain, plantar fasciitis, calcaneal spur

## Abstract

Feet are often the most neglected part of the body, all the while being the highly dependent part of daily work and mobility. The lack of attention to them can lead to painful conditions such as calcaneal spurs and associated conditions. Calcaneal spurs are bony projections that form around the calcaneal bone, the strongest, most significant, and posterior-most bone in the feet. The classic symptom of the calcaneal spur is talalgia, commonly known as heel pain. There are many causes of heel pain, which are usually associated with calcaneal spurs. Hence it becomes imperative to diagnose and treat them effectively. The development of calcaneal spur is shrouded in mystery, and why a few individuals are more prone to developing the condition than others depends on their gender, age, occupation, and lifestyle. Calcaneal spurs are seen in association with many diseases. It is also regarded as the etiological factor in plantar fasciitis and increasing body weight and as a complication in arthropathies, Gout, pes cavus, and pes planus. This review article aims to highlight a relationship between those factors while also summarizing the treatment modalities present today. Hence, it promotes the usage of a model for administering treatment based on a tier-wise follow-up procedure, where the response to a particular treatment is recorded. If it does not resolve the spur, the treatment progresses to the next tier. This review article hopes to shed light on the understanding and treatment of calcaneal spurs.

## Introduction and background

Calcaneal spurs are fibro-cartilaginous triangular projections that vary in size present on the calcaneum. There are two types of calcaneal spur based on their location, dorsal surface of the calcaneum is the dorsal calcaneal spur, and on the plantar surface, plantar calcaneal spur [1]. Plantar calcaneal spurs arise from the calcaneal tuberosity either from medial or lateral tuberosity. The calcaneum is the posterior pillar of the arch of the foot apart from being the largest, strongest and longest tarsal. It is the first foot bone to ossify [2]. It is related to the plantar fascia, dense connective tissue rich in fibrocytes. Plantar fascia is connected medially to the calcaneal tuberosity and extends to the digits of the foot. It maintains the arch of the foot and absorbs the tension created by weight bearing [3].

Traction of plantar fascia

According to this theory, the chronic traction at the insertion of the plantar fascia into the calcaneum leads to inflammation and subsequent ossification leading to enthesitis [4]. Studies have shown the relationship between increasing plantar fascial tension and the lowering of the medial longitudinal arch. There is also a positive correlation between the presence of heel pain and flatfoot [5,6]. This model of causation is not supported due to factors such as firstly, the direction of trabeculae is vertical- indicative of vertical compression; secondly, excision of the spur is followed by reformation and thirdly, there are no signs of inflammation seen at the site after histological examination in surgical excision [7,8].

Vertical compression

This hypothesis puts forth the pathology of micro-fractures, also called calcaneal stress fractures in the tendon, caused due to the repetitive compression of the plantar fascia. The formation of the fibrocartilaginous growths is a protective mechanism [9]. This corresponds to the association found in patients with increased weight and professions requiring long standing hours [10].

The heel pad and fascial thickness

According to a study, there is a gradual increase in the thickness of the heel pad with age and weight, which is accompanied by a decrease in the elasticity of the heel fascia. Hence the sub-calcaneal spur is directly associated with heel pain, also called talalgia [10]. Plantar calcaneal spur and fascial thickening are both established to cause plantar heel pain. However, their association is increased in the presence of both together. Plantar heel pain is a compound, multiple causative condition with varied tissue involvement [1].

Age variation

A study showed an incidence of plantar calcaneal spur of 11.2% and 9.3% of a dorsal calcaneal spur from a sample of 1026 lateral ankle X-rays [11]. Newer studies with larger and more age-appropriate samples, with lateral ankle X-rays of 1335 patients with a mean age of 46.5 years, showed an increased occurrence of either spur of 38% and both together of 11% [8]. The change in the gait of the geriatric age group is also a contributing factor to the increased incidence of the development of calcaneal spur. It is due to the heel and mid-foot contact time, reduced stride, and hence increased relative step count [3]. These studies also showed an increase in incidence and size of both calcaneal spurs types with an increase in age.

Gender variation

One study reviewed 1228 calcanei lateral X-rays with the frequency of plantar calcaneal spur as 14.6%, including 17.7% in females and 13% in males [12]. Conversation on the gender association of calcaneal spur is ongoing, with a few studies showing no gender disparities in occurrence, and other studies with a younger population showed an increased occurrence of the plantar calcaneal spur in females as compared to males and an increased occurrence of the dorsal calcaneal spur in males [13]. A gender difference was detected in only patients above the age of 50 when observed in cohort studies. The disparity may be due to wearing high-heeled shoes [14].

## Review

Associations of calcaneal spurs

**Figure 1:**
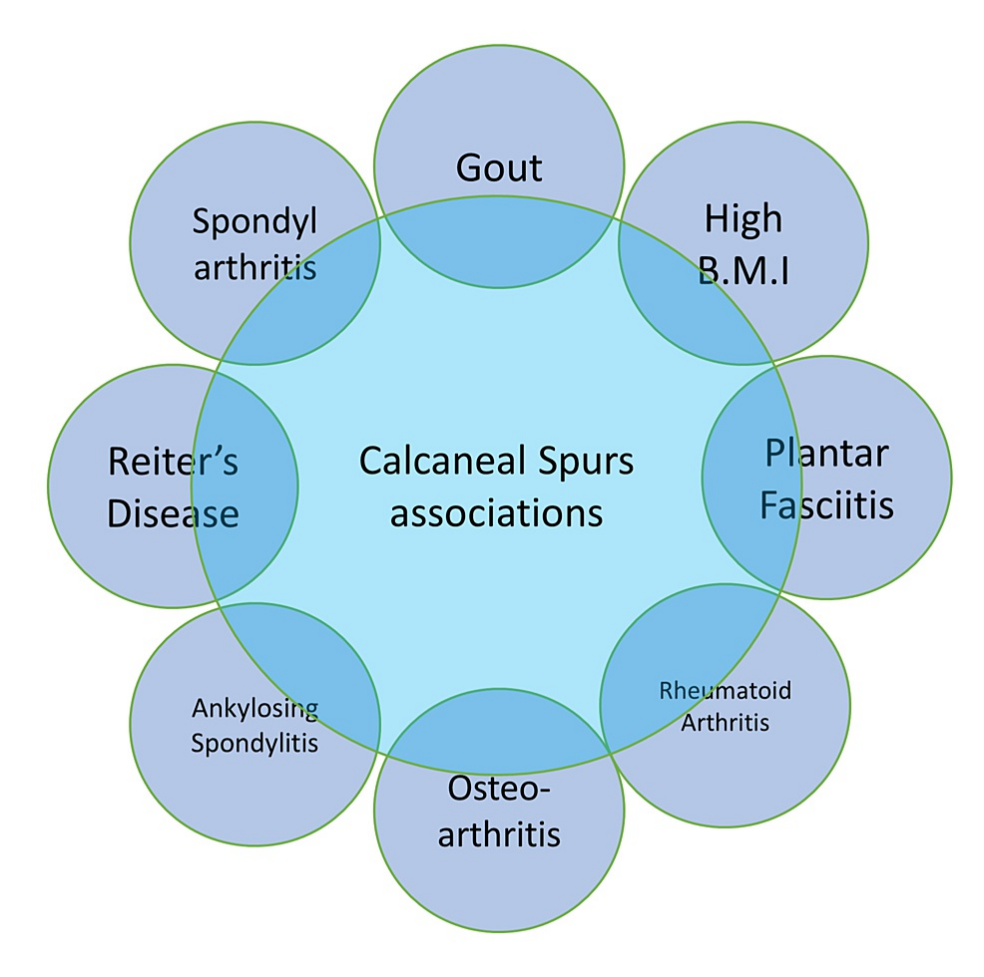
Associations of calcaneal spurs B.M.I.: Body mass index The figure is created by the author.

Calcaneal spurs occur in varied types of disorders, a few are shown in the Figure 1.

Gout

Gout is a type of inflammation in arthritis caused due to the gradual and chronic deposition of uric acid crystals. These crystals, which get deposited in the joints, cause severe pain, leading to inflammatory conditions in the affected joint. Deposition of these crystals in the joints serves as the diagnosis of gout. Sometimes the mono sodium urate crystals may also get deposited in soft tissues such as Achilles tendon. These conditions lead to the formation of calcaneal spurs at the site of insertion. A study also shows a significant relationship in patients with gout developing calcaneal, and Achilles spur. The study had a sample size of 181 patients. 44.7% of patients in the study showed the presence of either Achilles or plantar spur, and 22.1% of patients had both Achilles and calcaneal spur. The patients with either of the two spurs showed a greater association with high mean serum uric acid, metabolic comorbidities, duration after diagnosis of gout till the appearance of the spur, disease duration and hypertension. They concluded that the presence of comorbidities increased the disposition to development of spurs pertaining due to the pathology of faulty microvasculature and hypoxia, resulting in calcification on the site of insertion of the ligament and tendon [15].

Increased body weight

Obesity is the direct causative factor for the development of calcaneal spurs, with studies showing a positive correlation between increased body weight and calcaneal spurs conducted in military recruits [16]. Forty-five percent of the population had obesity and calcaneal spurs, compared to 9% who weren't obese. The concept of this association is backed by the theory of vertical compression in the development of calcaneal spur, which is affected by the altered gait of the person in obesity. Excessive body mass may cause the process of the increase in the heel pad and the toughening of the plantar fascia and even cause a faster age-related degeneration process [17]. Obesity has also been mentioned in a few studies as the cause of loss of the foot's medial arch, creating more traction and supporting the tractional theory of spur formation [9]. No clear causality exists between increased BMI according to studies but a higher degree of association with heel pain. There may be heel pain due to the increased BMI as the vertical forces are higher due to gravity, or there may be an increase in BMI because of heel pain [6,18].

Plantar fasciitis

From the literature reviewed, only one article said there isn't enough evidence to establish a connection to say that calcaneal spurs cause plantar fasciitis [3]. Other articles highlighted that there is a direct link between calcaneal spurs as one of the causative factors of plantar fasciitis [18-20]. Calcaneal spurs can be classified based on size, shape, and position in patients with plantar fasciitis. Plantar fasciitis is said to be a benign and self-limiting condition due to a degenerative process instead of an inflammation condition [18]. A study including thirty patients with heel pain who also had calcaneal spurs were treated surgically by removing the spur and the fascia removed, called endoscopic plantar fasciotomy, and the relationship between them was studied to classify the types of spurs. They classified them into two types, type A and type B. Type A was superior to the fascia, and type B was located within the fascia. Type B showed more severity in magnetic resonance imaging of plantar fasciitis before the surgical intervention. They also found a higher granulocyte count in type B rather than type A spurs. The patients did not show spur recurrence [19].

Rheumatoid arthritis, osteoarthritis, ankylosis spondylitis, spondylarthritis, and reactive arthritis

Studies show a frequent association of calcaneal spurs in patients with rheumatoid arthritis, ankylosing spondylitis, and Reiter's disease (Reactive arthritis). One of the studies showed that 21.6% of patients with rheumatoid arthritis had developed calcaneal spurs and 16% in controls owing to erosive property of rheumatoid arthritis and an increase in the incidence of osteoarthritis with increasing age [13]. Frequencies of each rheumatoid arthritis, Reiter's disease, osteoarthritis, and ankylosis spondylitis were calculated, and the highest incidence was seen in patients with Reiter's disease of 31% out of 16 patients with a complete triad (nongonococcal urethritis, arthritis, and conjunctivitis) for talalgias of moderate to severe type. Calcaneal spurs are the characteristic feature of Reiter's disease. The type of pain in Reiter's disease was more severe as compared to the other conditions [21]. Another study showed the association between spondylarthritis {SpA} and Achille's tendon enthesitis and bone erosion being separate processes, and SpA is related to the normal mechanic only. Still, these individuals showed smaller spurs at the initial stages of SpA and then larger spurs if the disease is chronic. The regions which were expected to undergo erosion showed a low bone-to-marrow ratio [22].

Clinical symptoms

Calcaneal spurs usually present as moderate to high heel pain, also called talalgia. Hence, the clinical symptoms are majorly pertaining to plantar heel pain. Plantar heel pain can arise from three broad origins. One is from the skeletal origin, such as calcaneal stress fractures, and inflammatory conditions. The second is soft tissue pathology, such as previously discussed foot fat pad atrophy, plantar fascia injury, and plantar fasciitis. Finally, heel pain may be induced because of neural causes such as compression of nerves, specifically Baxter's Nerve or lateral plantar nerve and medial calcaneal nerve, and tarsal tunnel syndrome [23].

Pain

According to studies, the patients with calcaneal spurs and plantar fasciitis reported early morning pain for 88% out of the study group of 250 patients. When they take their first steps, the pain gets relieved after walking almost half of them. The patients with foot pad atrophy had intense, severe pain, tingling, burning sensation, and cold. The pain usually increases after long walks, especially at night or after work while resting [23].

Foot anatomy and position

Ankle dorsiflexion was found to be painful and difficult in patients, the cause behind it being the biomechanical problem of heel cord tightness. There was an increased association seen in patients with pes planus, also known as flat foot, than patients with pes cavus [23].

Treatment

**Figure 2:**
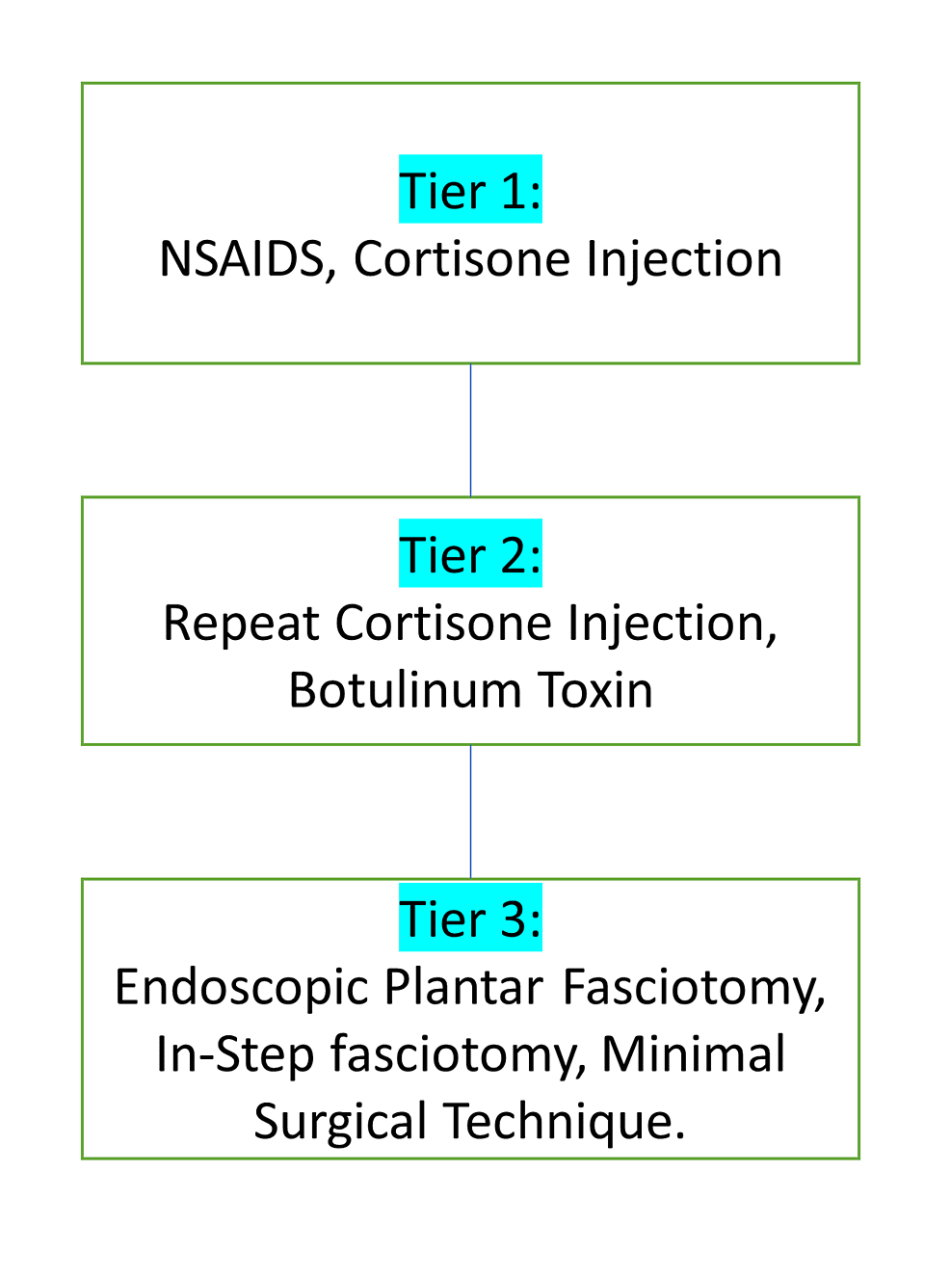
Medical management of calcaneal spur NSAIDs: Non-steroidal anti-inflammatory drugs The figure is created by the author.

**Figure 3:**
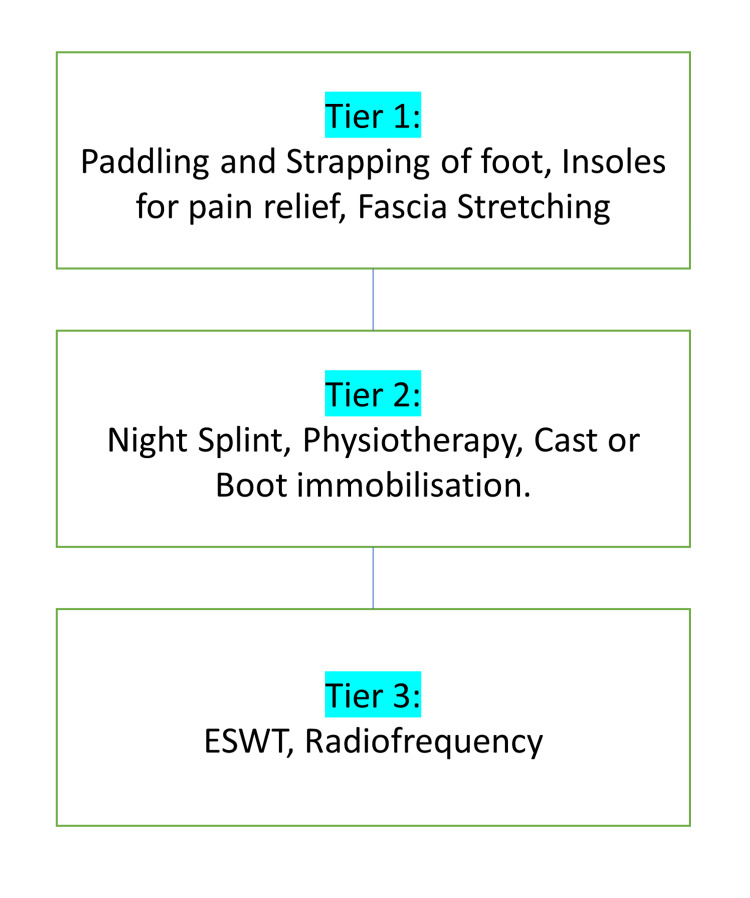
Conservative treatment of calcaneal spurs ESWT: Extracorporeal shockwave therapy The figure is created by the author.

There do exist many treatment modalities in research for calcaneal enthesophytes. Still, patients are usually known to suffer from the symptoms due to the lack of actual treatment modalities used clinically. This research aims to compile data for all the research treatment modalities present, to apply. Conventional treatment includes radiofrequency, ultrasound, laser, non-steroidal anti-inflammatory drugs, steroids, local anesthesia, extracorporeal therapy, splints, modified shoes with silicon shoe implants, physiotherapy, stretching, and taping (Figures 2, 3) [24].

Gastrocnemius-soleus stretching over other stretching methods

A study found the increased effectiveness of gastrocnemius-soleus stretching in patients aged 30-70 suffering from painful heel spur syndrome. It established that gastrocnemius-soleus stretching is more effective than Tendo-Achille's stretching, as the group of patients who practiced gastrocnemius-soleus stretching showed a significant amount of reduction in pain score than what was the initial pain score before intervention. The stretching of gastrocnemius and soleus muscles causes an increased range of dorsiflexion movement. This stretching relieves rigidity or lack of flexibility due to excessive pronation and the overcompensation of the first metatarsal joint's plantar fascia. This inflexibility also caused increased tension at the calcaneum insertions. Stretching is a type of conservative treatment for heel spur. This type of therapy is cost-effective and patient-centric; hence may become a better form of therapy in the long run [25].

Extracorporeal shock wave therapy and laser treatment therapy

Studies show that extracorporeal shock wave therapy has been effectively used to treat patients with calcaneal enthesophytes. They do so by using shocks of 0.03mJ/mm2 energy applied, which is gradually increased to 0.4mJ/mm2 since the patient may experience pain at the start. These variations in symptoms were measured using the visual analogue scale {VAS} [26]. Other diagnostic tests used were X- rays for dimension monitoring throughout the treatment and grade of enthesis by sonography [27,28]. These studies concluded that extracorporeal shock wave therapy was a safe treatment modality because the results showed a significant decrease in the visual analog scale of about 68% along with morphological changes on X-rays, but there were no changes in grades observed on sonography [24]. Another study was based on the efficacy comparison of extracorporeal shock wave therapy and laser treatment therapy. Two groups were subjected to different treatment modalities, and the results were evaluated based on the visual analog scale. There was no significant difference in their score [29]. Hence establishing both are equally good treatment modalities for calcaneal enthesophytes.

Cryo-ultrasound therapy

A study of a sample size of 102 patients with chronic plantar fasciitis and heel spurs debated the effectiveness of using cryo-ultrasound therapy or using just cryotherapy. They used different types of treatment on each subject group and evaluated their visual analog score. Treatment was administered 10 times, each lasting for 20 minutes. The patients were evaluated at time intervals of three months, 12 months, and 18 months. There was a difference of 3.00, 4.35, and 4.81 points, respectively, in the score observed after each follow-up in the group who had undergone cryo-ultrasound. Hence after reviewing the effectiveness index of both, a better response was seen in patients who had undergone cryo-ultrasound with long-lasting effects in relieving plantar fasciitis [30].

Radiological treatment

Various studies are present on radiological treatment as it is one of the least invasive therapy and is emerging as one of the most effective therapy with the least side effects. The effectiveness of the therapy is measured in terms of the reduction of pre-radiation pain [31]. In cases of intralesional radiofrequency treatment is used when they are not responding to anti-inflammatory drugs and corticosteroid injections or other mechanical management such as shoe insoles [32]. A low dose of radiation is used, and after compiling the articles under study, the frequency used ranges from 3-12 Gy with less or almost no side effects [24,33].

Surgery

The failure of conservative therapy leaves only surgical intervention, which can only be done after 9/12 months. The operations to treat plantar fasciitis due to calcaneal spur involve endoscopic plantar fascial resection, the most common surgical process done. It may be done along with calcaneal spur resection or alone. Both processes have been known to be effective. Endoscopic and fluoroscopic calcaneal spur resection without plantar fascial release also had favourable outcomes. The older literature shows another approach to creating a workspace while resectioning the plantar fascia and calcaneal spur. This creates a more invasive approach than endoscopic surgery. The post-operative care in the endoscopic approach is much faster than in other surgeries [34,35].

## Conclusions

Calcaneal spurs, also known as calcaneal enthesophytes, is a condition whose cause has long been debated. This article intends to provide a summary of the theories put forth previously to hone in on the exact etiology and mechanism of the formation of a spur. The majority of cases reported have been theorized under vertical compression theory, which corresponds to the high association of increased BMI. After reviewing the literature, we found that there was also an increase in incidence seen with progressing age, which was also accompanied by cases of gout and other arthropathies in older patients. The patients are known to experience heel pain, also known as talalgia, throughout their life without seeking effective treatment. The new treatment modalities have been researched to find their effectiveness and their application to become standardized. Currently, there is a surgical procedure that shows the lowest rate of reformation of the spur and safer treatment, but it can only be done after 9-12 months. This research highlights the fact that there is a need for a treatment modality other than conservative treatment for the period before 9-12 months to reduce and alleviate the patient's condition suffering in pain. Extracorporeal shock and laser treatment are one of the best currently in study to become standardized for clinical practice. Calcaneal spurs cause many lifestyle changes, associated as a cause of increased BMI, affect heavy lifting, create inconvenience to a pregnant mother, and adversely affect the performance of athletes. It may become debilitating in patients for whom even walking may become a challenge worsening the condition itself. It also affects the footwear the patients need to use. This article puts forth the need to create a treatment and management paradigm against the wait-and-see approach in the calcaneal enthesophytes background.
